# Combining app-based behavioral therapy with electronic cigarettes for smoking cessation: a study protocol for a single-arm mixed-methods pilot trial

**DOI:** 10.1186/s13722-024-00483-5

**Published:** 2024-07-10

**Authors:** Helen Schiek, Tobias Esch, Maren M. Michaelsen, Cosima Hoetger

**Affiliations:** Institute for Integrative Health Care and Health Promotion (IGVF), Faculty of Health/School of Medicine, Witten/Herdecke University, Witten, Germany

**Keywords:** Smoking cessation, Digital health, mHealth, Cognitive behavioral therapy, Mindfulness, Electronic cigarettes

## Abstract

**Background:**

Cigarette smoking remains a leading cause of preventable illness and death, underscoring the need for effective evidence-based smoking cessation interventions. Nuumi, a novel smoking cessation program integrating a digital behavioral therapy and an electronic cigarette, may provide a solution.

**Objective:**

To investigate the initial efficacy, acceptability and psychological outcomes of an evidence-based smoking cessation intervention comprised of a mobile phone app and an electronic cigarette among adults who smoke and who are motivated to quit.

**Methods:**

A prospective 6-month single-arm mixed-methods pilot study will be conducted. Seventy adults who smoke and who are motivated to quit will be recruited via web-based advertisements and flyers. Participants receive access to an app and an electronic cigarette with pods containing nicotine for temporary use of at least 3 months. The electronic cigarette is coupled with the app via Bluetooth, allowing for tracking of patterns of use. The behavioral therapy leverages evidence-based content informed by cognitive behavioral therapy and mindfulness-informed principles. Web-based self-report surveys will be conducted at baseline, at 4 weeks, at 8 weeks, at 12 weeks, and at 24 weeks post-baseline. Semi-structured interviews will be conducted at baseline and at 12 weeks post-baseline. Primary outcomes will be self-reported 7-day point prevalence abstinence from smoking at 12 weeks and 24 weeks. Secondary outcomes will include other smoking cessation-related outcomes, psychological outcomes, and acceptability of the nuumi intervention. Descriptive analyses and within-group comparisons will be performed on the quantitative data, and content analyses will be performed on the qualitative data. Recruitment for this study started in October 2023.

**Discussion:**

As tobacco smoking is a leading cause of preventable morbidity and mortality, this research addresses one of the largest health burdens of our time. The results will provide insights into the initial efficacy, acceptability, and psychological outcomes of a novel mobile health intervention for smoking cessation. If successful, this pilot may generate an effective intervention supporting adults who smoke to quit smoking. The results will inform feasibility of a future randomized controlled trial.

*Trial Registration* German Clinical Trials Register DRKS00032652, registered 09/15/2023, https://drks.de/search/de/trial/DRKS00032652.

## Introduction

Worldwide, tobacco use constitutes a leading cause of preventable illness and death [1]. A variety of evidence-based psychological and pharmacological interventions are available to support cessation, including brief behavioral counseling, telephone counseling, behavioral therapy in one-on-one or group settings, nicotine replacement therapy (NRT), or pharmacotherapy such as varenicline, bupropion, or cytisine [2]. However, these treatments are rarely utilized by adults who smoke to support cessation [3], and even when used, long-term abstinence rates tend to be relatively low, often falling below 15% at 6 to 12 months after the intervention [4]. Hence, new and effective treatment approaches for adults who smoke and who are willing to quit smoking are needed.

A drawback of conventional treatments such as NRT products is that, while they may effectively replace nicotine intake, they do not fully address the sensory, behavioral, and social characteristics of smoking; including activities like holding a cigarette, taking puffs, deriving pleasure from smoking, or feeling part of a social group while using them [5]. A method that may circumvent these limitations is the use of electronic cigarettes (ECs).

### Electronic cigarettes for smoking cessation

ECs constitute a novel and diverse product class of electronic devices comprised of a reservoir holding a liquid solution, a power source (e.g. battery), and a heating element [6]. The liquid solution contains solvents such as vegetable glycerin and/or propylene glycol, flavorings (e.g. tobacco, menthol) and may contain nicotine [6]. Although not officially approved as a smoking cessation aid in most countries, like Germany [2] or the U.S. [7], ECs are the most popular smoking cessation tool in Germany [3], and in the U.S., almost half of adults who smoke and want to quit indicate they had tried ECs [8].

The use of ECs has been linked to significantly greater smoking abstinence rates relative to non-supported quitting attempts [3], and there is high-certainty evidence available that nicotine-containing ECs support smoking cessation more effectively than NRT, and moderate certainty evidence that they increase quit rates compared to ECs without nicotine [5, 9]. The relative effectiveness of ECs may be partially explained by the higher reduction in withdrawal symptoms relative to NRT products [10]. The degree to which ECs can effectively suppress nicotine withdrawal symptoms varies across devices [11, 12], and nicotine delivery is dependent on various factors, including device power [13], nicotine concentration [14], and user experience [12]. ECs deliver some of the sensory and motoric components of cigarette smoking, e.g. hand-to-mouth movement and “throat hit” [5, 15]. Therefore, ECs may have the potential to alleviate undesirable nicotine withdrawal symptoms while addressing both the behavioral aspects, rituals and sensations associated with smoking [5]. In fact, even non-nicotine-containing ECs have been linked to suppression of some withdrawal symptoms [12].

Another advantage of ECs is that they allow for individual tailoring of nicotine doses [10] and hence may be used to reduce nicotine over time. In combustible tobacco cigarettes, reducing nicotine content has been linked to a reduction in the relative reinforcing effects of smoking, a key marker of dependence potential [16].

However, although evidence suggests that ECs are likely to be less harmful than combustible cigarettes, they are not without risk, and concerns remain regarding the long-term safety of their usage [17]. While ECs may serve as an effective smoking cessation tool, meta-analysis data shows that approximately 70% of individuals who underwent EC-supported smoking cessation report ongoing EC use at six months or longer [18]. Because the long-term health consequences of ECs use are unknown, EC-supported smoking cessation efforts should include steps to wean individuals who had previously smoked off ECs once smoking cessation has been attained.

In summary, ECs can serve as effective smoking cessation tools by providing individuals with nicotine to suppress withdrawal symptoms. However, individuals undergoing a smoking cessation attempt also suffer from increased affective symptoms such as depressive or anxious mood, irritability, and restlessness [19–21], and factors such as an encounter with other adults who smoke or experiencing stress may further exacerbate the risk of relapse [22, 23]. Thus, to provide adults who smoke with a successful smoking cessation intervention, a program should include components that address the psychological mechanisms of addiction in addition to physical symptoms.

### Behavioral interventions for smoking cessation

Evidence suggests smoking cessation efforts are more effective when behavioral support is offered in addition to NRT interventions [24, 25]. Effective behavioral treatments include brief cessation counseling, telephone support, text messaging interventions, mobile application, or webpages, and face-to-face individual or group-based cognitive behavioral therapy (CBT) [26] with the latter having been shown to be more effective than brief advice, self-help materials, or counseling [26].

CBT is a psychological treatment approach that aims to help individuals quit smoking by modifying thoughts, beliefs, and behaviors associated with smoking [27]. CBT-based interventions often target problem solving and coping skills, teaching strategies like cognitive restructuring of maladaptive thoughts [27–29]. As a further development of CBT, mindfulness-informed interventions (MIIs) have emerged within the so-called third-wave generation of CBT treatments [30, 31]. In MIIs, conventional CBT methods are enriched with mindfulness- and acceptance-based strategies as well as metacognitive and meditative components [2]. Mindfulness typically involves intentionally directing one’s attention toward physical sensations, emotions, and thoughts to cultivate a heightened awareness and foster a nonjudgmental acceptance of these inner experiences [32]. Mindfulness training usually includes training of attention regulation, body awareness, emotion regulation, and self-awareness among other skills [33, 34]. In smoking cessation, mindfulness training teaches individuals the skill to act as an observer of one’s own affective state and cessation-related cravings instead of reacting by resorting to smoking [35]. While a recent Cochrane review was unable to detect clear benefits of mindfulness training for smoking cessation when compared with other smoking cessation treatment or no treatment [36], some literature has pointed out that adding a mindfulness component may increase the likelihood of quitting smoking [37]. Importantly, mindfulness seems to act on factors related to smoking and may therefore indirectly impact smoking abstinence. For example, mindfulness training may aid in reducing the number of cigarettes smoked per day [38], weaken the link between cigarette cravings and smoking [39, 40], and enhance the self-efficacy of adults who smoke for managing negative emotions without using cigarettes [41].

However, while adding psychological components to smoking cessation interventions can generally increase intervention effectiveness, in-person counselling is costly and is difficult to access for some due to limited financial resources and time. While in-person interventions may not present a feasible and scalable treatment for all adults who smoke and are willing to quit, digital behavioral therapies provide an easily accessible alternative. Mobile health (mHealth) interventions like smartphone apps offer numerous advantages over traditional in-person behavioral smoking cessation support, such as improved accessibility and availability, real-time support, personalization of features, cost-effectiveness, and scalability [42–44].

Currently, there are few high-quality studies available examining the effectiveness of smartphone apps for smoking cessation [45], and the content of many currently available app-based programs is only marginally consistent with evidence-based treatment guidelines [44, 46]. However, in principle, digital therapeutic interventions offer opportunities for intensive therapeutic programs and recent studies suggest that such interventions can effectively support smoking cessation [42, 45, 47, 48]. Importantly, a growing body of literature highlights the effectiveness of digital CBT-based programs for smoking cessation [42, 49], and MIIs have also shown initial efficacy when delivered via mHealth [50].

The aforementioned findings highlight mHealth applications as a promising tool for providing effective behavioral smoking cessation interventions. However, while an increasing number of smoking cessation apps are tailored to be administered alongside NRT, to our knowledge, there is no mobile application available supporting adults who smoke and want to quit by using ECs.

Considering the findings that some ECs can support smoking cessation more effectively than NRT, and smartphone apps provide the opportunity to offer behavioral support to adults who smoke and are willing to quit, Sanos Group GmbH (Berlin, Germany) developed an evidence-based smoking cessation program featuring an integrated EC and app-based behavioral therapy, *nuumi*. This study will be the first trial to evaluate the intervention.

### Objectives

The primary objective of this study is to assess initial efficacy of the nuumi program, defined as self-reported 7-day point prevalence abstinence from smoking at 12 weeks and 24 weeks after initiation. Secondary objectives are to examine within-group changes in smoking cessation-related and psychological outcomes, including urges to smoke, perceived stress, mindfulness, self-efficacy to abstain from smoking, subjective health, and life satisfaction. A further secondary objective is to investigate the acceptability of the nuumi program, indexed by perceived usefulness in quitting smoking, user satisfaction, comprehensibility of the content, and usability of the app. The objective of the qualitative interviews is to explore the perspectives of adults who smoke on quitting using electronic cigarettes and apps before the intervention, and their perspective about the intervention after completion. Overall objective of this single-arm trial is to pilot and optimize procedures of a subsequent RCT, in which the nuumi intervention will be compared to a treatment-as-usual (TAU) control group.

## Methods

### Study design and setting

This study is a prospective 6-month, single-arm mixed-methods pilot study designed to evaluate smoking cessation outcomes, acceptability, and psychological outcomes of the mHealth intervention nuumi. Data will be collected in Germany.

### Participants

Interested individuals are screened for eligibility by completing a brief online questionnaire. Eligible participants are aged 18–65 years, report having smoked at least 5 cigarettes per day (CPD) for at least 12 months, are motivated to stop smoking (Motivation To Stop Scale (MTSS; [51]) > 4 points), have daily access to their own smartphone (iOS 15/Android 11 or more recent), reside in Germany, have access to a personal email account, and report being able to read and write in German.

Ineligibility criteria include self-reported current or planned pregnancy, breastfeeding, a self-reported allergy to vegetable glycerin or propylene glycol, drug and/or alcohol dependence, severe psychiatric or physical illness, a disease or medication associated with a contraindication to the use of EC, and medication that could affect the outcomes of the study (bupropion/ nortriptyline/ varenicline/ cytisine/ clonidine/ antidepressants). Exclusion criteria also include surgery (with anesthesia) in the last 6 weeks, participation in any other smoking cessation program, current use of EC/tobacco heaters/alternative tobacco products/NRT for more than 5 days during the last 30 days, and the inability to consent.

### Recruitment

Recruitment started in the end of October 2023 via online advertisements, flyers, and a study website. A total of 70 participants will be recruited until recruitment is complete. Interested individuals can register on the study website and participate in a screening survey. Participants who are deemed eligible by study staff will be informed of their eligibility and receive the informed consent document. The document contains detailed information about the intervention, study procedures, potential risks and benefits of study participation, data handling and data protection. Consent can be given online by clicking on a “I consent” checkmark box. Upon submitting their informed consent form (Appendix B) electronically, participants will be emailed an electronic link to the baseline survey (t_0_). To prevent individuals from participating in the survey more than once, study staff will screen for duplicates of first and last names and email addresses during registration.

### Incentives

Participants will receive 10€ for each completed survey (t_1_- t_4_). For participation in the semi-structured interviews, participants can earn another 10€ for each interview for a total incentive of 60€ once the study has ended.

### Participant timeline

The study will be conducted over a six-month period, during which a total of five surveys will be administered and two interviews will be conducted with a subgroup of participants. Quantitative data collection will take place at t_0_, 4 weeks post-baseline (t_1_), 8 weeks post-baseline (t_2_), 12 weeks post-baseline (t_3_) and 24 weeks post-baseline (t_4_). All participants will receive access to the nuumi intervention directly after t_0_ data collection. Participants will be granted 2 weeks to complete each survey. Primary and secondary outcomes of this study will be answered by analyzing t_3_ and t_4_ data. To minimize fraud potential, survey invitations are personalized and can only be accessed with an individual access key. Surveys can only be completed once by using the individual access key.

Semi-structured interviews will be conducted by research staff with a sub-sample of 15 enrolled participants via videocall or telephone at t_0_ and t_3_. Participants are invited to partake in the baseline survey. Interested participants will indicate their willingness to be invited via email at the end of the baseline survey and will be sent an informed consent form to be signed electronically (Appendix B). The first interview will be conducted within 2 weeks after t_0_, and the second interview within 2 weeks after t_3_. In interview 1, participants will be asked about previous cessation attempts and perceived barriers to smoking cessation, their perceptions, and attitudes towards ECs and towards the use of smartphone apps for smoking cessation. In interview 2, participants will be interviewed about their current smoking and EC use status, their perceptions of the different intervention features, and the perceived effectiveness of the program. Participants may participate in both interviews, however, having participated in the first interview is not a prerequisite for participation in interview 2.

**Fig. 1:**
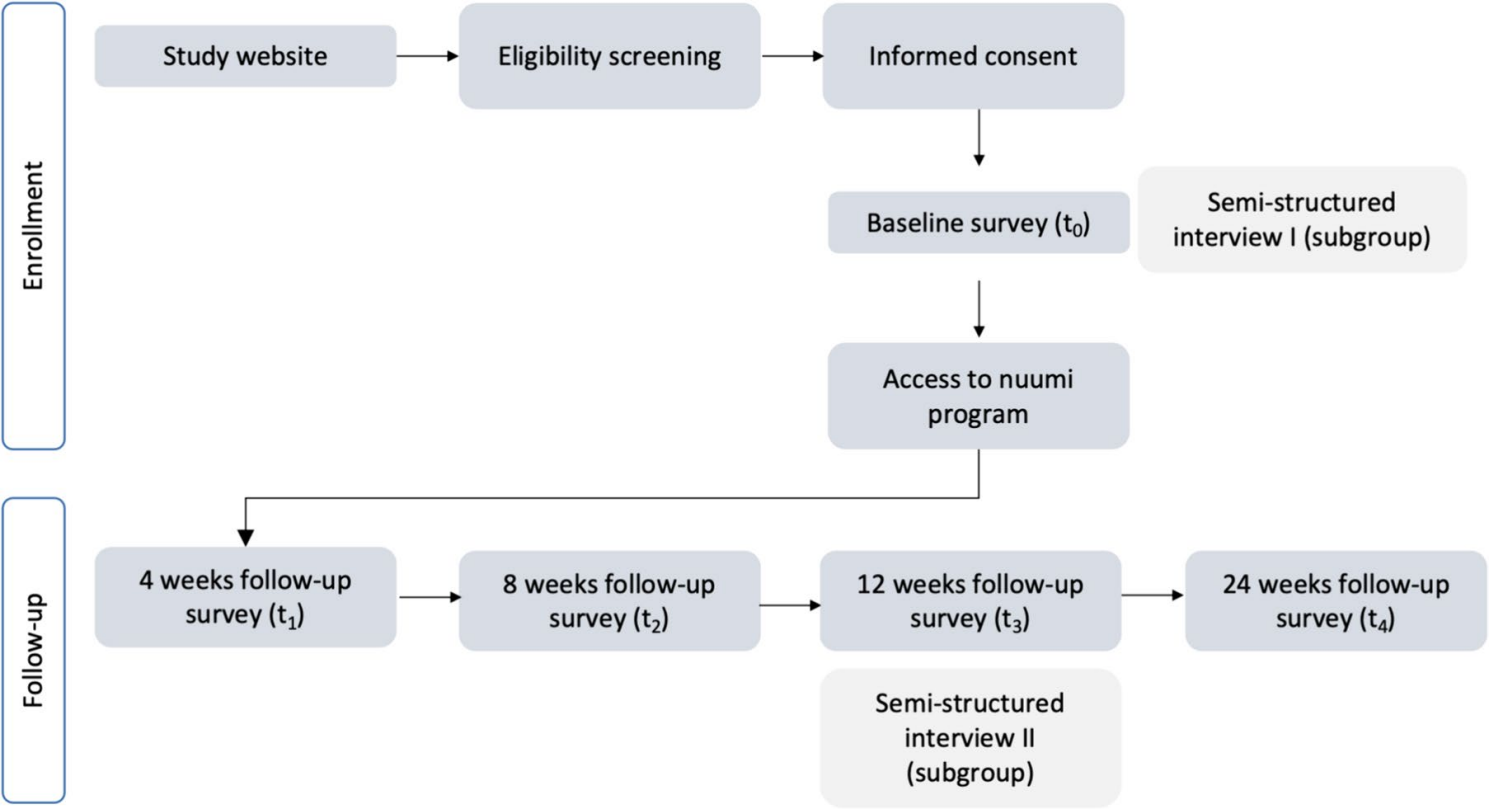
Participant flow and study design

Participant flow and study design are outlined in Fig. 1 in an adapted Consolidated Standards for Reporting Trials (CONSORT-EHEALTH) diagram for pilot and feasibility trials [52, 53].

### Intervention

Nuumi is a self-guided digital therapeutic intervention comprising an app-based behavioral therapy and an EC connected to the app via Bluetooth. Initially, participants are asked to use the EC whenever they crave a cigarette, hence replacing cigarettes with the EC. In parallel, participants are prompted to use the app-based behavioral therapy providing information on transitioning to the EC, and smoking cessation. The app also contains information on gradual EC cessation, thus supporting the user in achieving abstinence of both products.

After completion of the baseline survey, participants receive instructions to download the app and will receive a voucher allowing them to order the EC and pods lasting for a minimum of 3 months from the manufacturer’s website at no cost. Participants are also issued a prepaid return label to return unused pods to the manufacturer in case they decide to stop using the EC and/or the program altogether.

Participants are not required to quit smoking immediately after baseline; they are advised to switch from combustible cigarettes to using the EC either by choosing a quit date, or by gradually switching from smoking to using EC over a 2-week period. The EC has been developed and manufactured by the funder of this study. It is a closed system device; i.e. empty pods cannot be refilled by the user and must instead be replaced with prefilled pods obtained through the manufacturer which can only be used after activation via the app.

Participants will receive a kit including the EC, a charger, a power bank, pods, and manuals for the EC and the pods via mail. Each participant will be supplied with an amount of pods equivalent to their respective cigarette consumption at study entry, estimated according to a guideline by the manufacturer. Participants can choose between two kinds of tobacco flavors when ordering the EC. The liquid solution in the pods includes propylene glycol, glycerol, nicotine, and flavoring. The EC is puff activated and the settings cannot be modified by the user. It is powered by a 450 mAh battery.

The participants receive pods in nicotine strength 20 mg/ml to 0 mg/ml, decreasing in steps of two mg/ml. Participants are prompted to start out with the 20 mg/ml pods and gradually use pods containing lower nicotine strength when one has been used up.

**Fig. 2:**
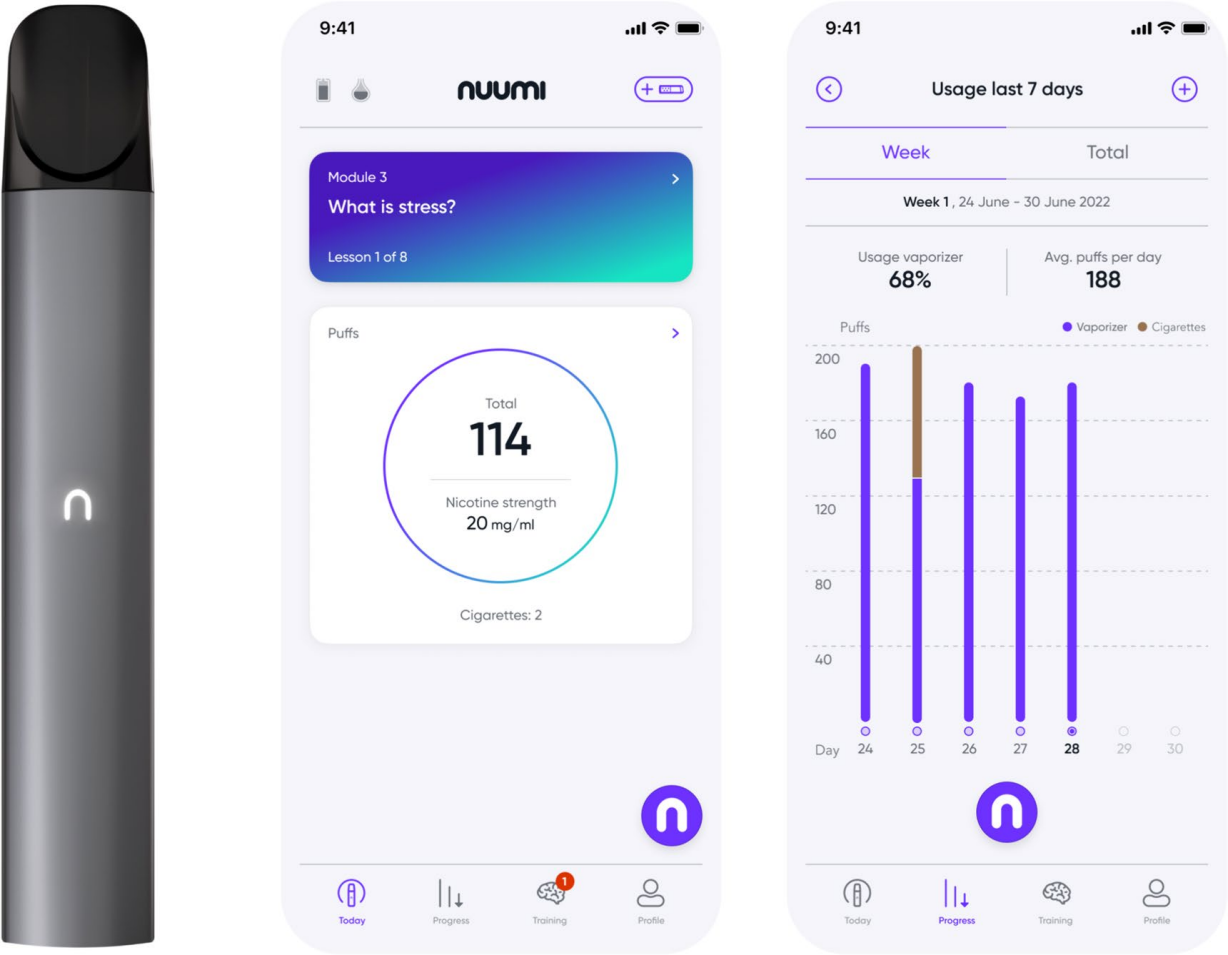
(1) Bluetooth-enabled nuumi EC, (2) app section *Today* depicting tracking of daily puffs, current nicotine strength, and tracked cigarettes, (3) app section *Progress* showing EC use statistics

The EC is coupled with the nuumi app via Bluetooth, allowing for tracking of patterns of use, e.g. number of daily puffs (see Figure 2). These data are made visible to the user in the app. Two weeks after program start, users are presented a daily puff budget based on their average daily puffs previously recorded and are encouraged not to exceed the number of allotted puffs. Limiting the number of puffs serves the purpose of preventing compensatory puffing [54] when the nicotine concentration in the liquids is reduced. An app feature allows participants to reduce or increase their puff budgets as needed.

**Fig. 3:**
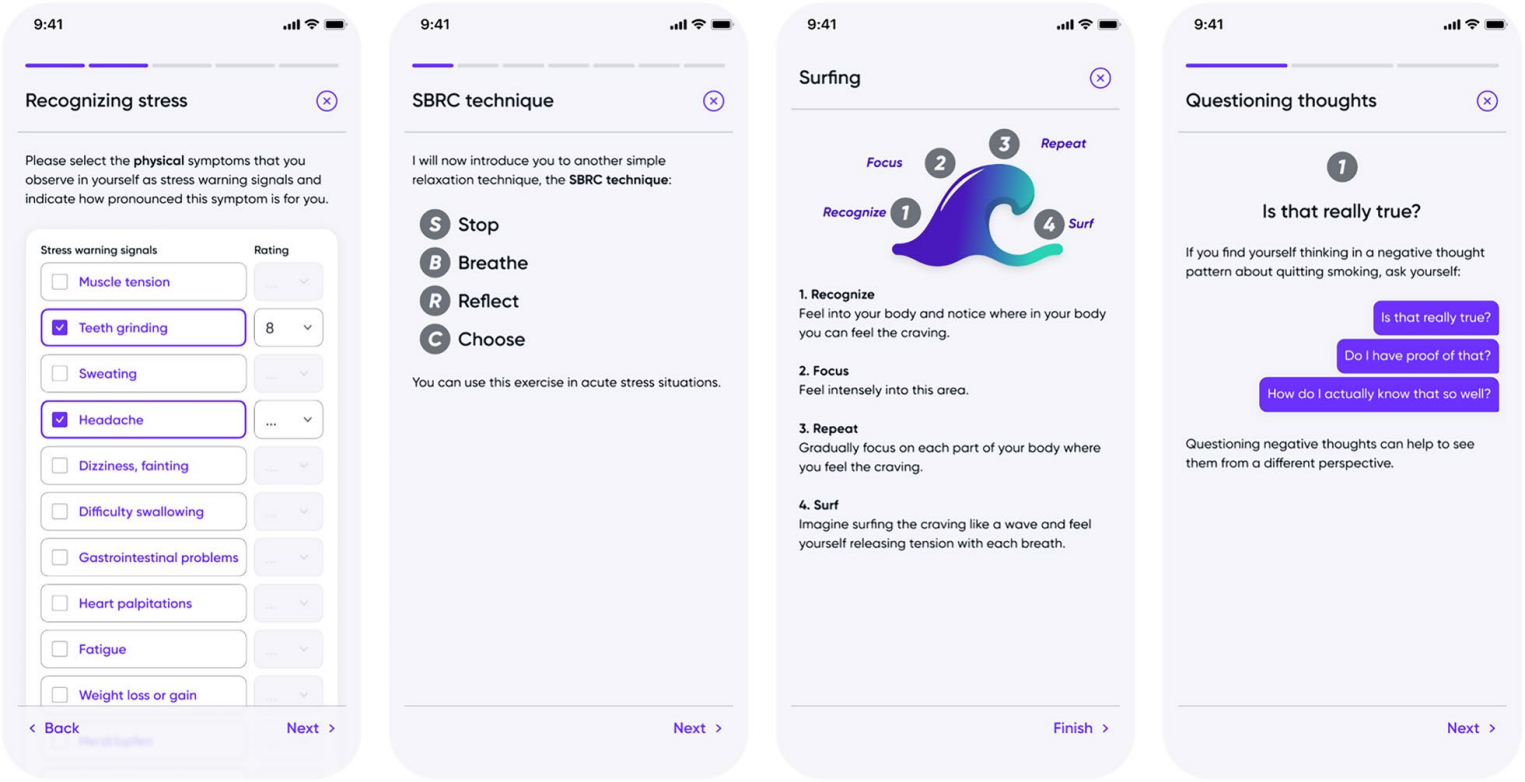
Examples of lessons within the therapy modules

To support participants in transitioning to the EC, and to subsequently reach abstinence from the EC, behavioral therapy is provided in the nuumi app which features evidence-based content informed by CBT-based and mindfulness-informed principles. Basis of the behavioral therapy is an initial in-person health promotion and stress management course certified by German statutory health insurance which features information from four core areas, including behavior, exercise, relaxation, and nutrition (BERN, [55, 56]). For the purpose of the present smoking intervention, the course has been digitalized, and its content has been modified to meet the needs of adults who smoke. The content is delivered via educational audio recordings, interactive exercises, and quizzes. The user is prompted to complete a total of 11 modules with each new module made accessible after the previous one has been completed (see Table 1 Appendix A and Fig. 3).

**Fig. 4:**
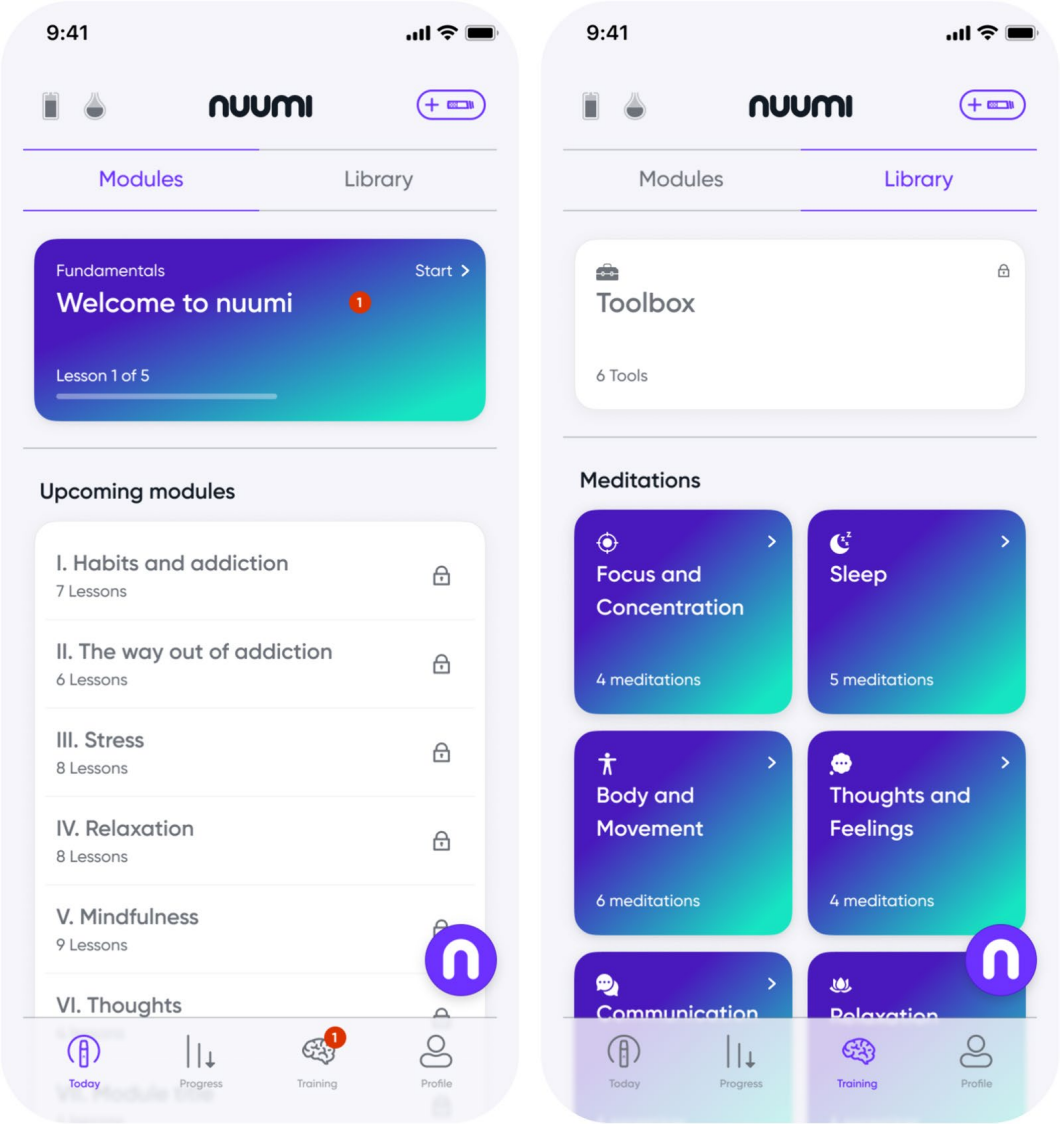
App section *Training* consisting of (1) 11 therapy modules, and (2) a *Library* featuring a *Toolbox* and meditation audios

Abbreviated versions of the coping techniques introduced in the modules are made accessible within the *Toolbox* section of the app; participants are advised to apply these coping techniques when dealing with cravings, stress, negative thoughts, and emotions (e.g. “urge surfing” [57]). In addition, the app features a meditation library with 32 guided meditation audios (see Fig. 4). The meditations, recorded by a professional meditation speaker, are split into 8 categories (relaxation, thoughts and feelings, sleep, body and movement, focus, communication, compassion and gratitude, happiness) and are accompanied by binaural beats [58].

As a means of self-monitoring their cessation behavior, users can track the number of smoked combustible cigarettes in the app. Twelve puffs per cigarette are added to their daily EC puffs and depicted accordingly in the app. Additional nuumi app features include a dashboard that participants can use to track their current pod nicotine concentration, number of daily puffs, as well as progress on the behavioral therapy modules, and meditation minutes. Further, as a self-monitoring tool, users are prompted to answer one question concerning their smoking self-efficacy once per week within the app (“How confident are you that you will be completely abstinent from smoking cigarettes in one year from now?” not confident at all—very confident). Daily check-ins take place throughout the first 14 days of the nuumi program. During these check-ins, participants learn more about EC use and receive tips and motivational content to aid their transition from cigarettes to the EC. Users receive daily push notifications containing motivational and informative text messages based on the therapy modules. No substantial revisions to the app are planned during the trial period, and the anticipated app updates are limited to bug fixes.

The nuumi intervention was developed within the framework of Michaelsen and Esch’s Behavior Change Resource Model (BCRM) [28, 59]. The BCRM states that in the development of effective interventions for health behavior change, it is crucial to identify the resources necessary for successful behavior change and to select behavior change techniques (BCTs) most likely to support individuals in reaching their target behavior. Drawing upon a range of theories from social and health psychology, the behavior change process is commonly characterized as involving distinct stages (non-awareness, awareness, contemplation, planning, initiation, continued action, maintenance) that individuals traverse in pursuit of their goal [60]. At each of these stages, individuals require specific resources, defined as factors of an individual that can affect behavior, which can be categorized into three distinct sets, i.e. external, internal reflective, and internal affective resources which are characterized by both changeable and non-changeable factors [28, 59].

Internal resources are defined as bio-psychological resources pertaining to an individual’s internal state. *Internal reflective* resources are generated, accessed, or improved through effort and conscious deliberation, such as goals and behavioral regulation [28]. *Internal affective* resources, in contrast, can quickly be activated through stimuli without intentional effort, e.g. emotions and their reinforcement [28]. *External* resources refer to the socio-environmental resources that facilitate behavior change and can be provided, modified, or generated by the individual or others [28]. All these resources can be addressed by health BCTs, categorized into three groups: facilitating, boosting, and nudging [28, 59].

*Facilitating* BCTs provide external resources to facilitate new behavior, such as changes in the environment or material resources [28, 59]. The behavior change initiated through facilitating BCTs can, by definition, only last as long as the provision is sustained. However, through the provision of external resources, individuals may acquire a set of skills or routines to sustain the new behavior even when the external support is no longer available [28, 59]. A facilitation-based feature of the nuumi intervention is for example the provision of the EC as a material resource. By gaining access, individuals may acquire skills allowing them to navigate a situation in which they are offered a cigarette; once adopted, the skill can be applied even if the EC is no longer available [28, 59]. *Boosting* BCTs aim to strengthen internal reflective resources [28, 59]. By incorporating cognitive involvement of the individual, boosts foster competencies through changes in skills, knowledge, or decision-making competencies [61]. Boosting-based features included in the nuumi program are e.g. educational videos, audios and interactive exercises within the behavioral therapy part. Theoretically, the strengthening of said resources should generate persistent effects even after the intervention is removed, as competencies including dependence-related knowledge or mindfulness can remain stable over time [28, 59]. Lastly, *nudging* BCTs guide behavior by modifying choice architecture to a specific direction while maintaining freedom of choice [28, 59]. By intentionally applying stimuli, cues, or triggers in an environment, nudging engages affective decision-making elements, rendering the desired behavior more motivationally appealing and intrinsically rewarding [28, 59]. Nudging interventions do not rely on cognitive skills or the provision of external resources to be effective, but rather function through affective components of human motivation and reward processes [28, 59]. These interventions can lead to the formation of new routines without intentional effort from the individual, particularly when they are repeatedly applied [28, 59]. Nudging-based features of nuumi include, among others, the progress tracking dashboard, and daily push notifications containing motivational text messages.

Informed by the BCRM [28, 59], nuumi’s features make use of facilitating, boosting, and nudging techniques to produce behavior change among adults who smoke and are motivated to quit. BCTs were carefully selected and applied, tailoring nuumi to meet their specific needs.

### Data collection, management, and monitoring

Data will be collected via a web-based data collection tool (*LimeSurvey*; LimeSurvey GmbH, Hamburg, Germany). Participants will receive a personalized link to the web-based survey via email and will receive up to two reminder mails within a period of two weeks prompting them to complete the surveys. Data from non-completers will be used to calculate the loss to follow-up rates. Participants will be allowed to skip any question they do not wish to answer. At the end of each web-based survey, the participants can enter feedback or additional comments into a text box.

The university is responsible for storing and protecting the research data. All data will be stored in electronic format on a secure university network location accessible only by authorized members of the research team. The data monitoring committee will comprise the university research team. During data collection, range checks for data values and additional steps to ensure data quality will be conducted by two research team members. Preliminary analyses are planned for t_1_, and t_2_ data after data collection of the respective time point is completed. Primary and secondary outcomes will be assessed with t_3_ and t_4_ data to address the aims of this study. Qualitative data from semi-structured interviews at t_0_ and t_3_ will be analyzed upon completion of all interviews.

App feature utilization data collected by Sanos Group GmbH is stored on a secured network location only accessible by employees of Sanos Group GmbH. Sanos Group GmbH receives the name and email address of the participants for the transmission of the app usage data to the research staff. The participants consent to this data exchange in the informed consent document.

Adverse events will be reported to the Institutional Review Board (IRB) and to the study funder. Documentation of such events will be stored on the university’s secure network location. Participants reporting severe adverse events or side effects from the EC use will be suggested to not use the EC further.

### Outcomes

Outcome measures and other variables with their respective assessment time points are shown in Table 2 in Appendix A.

#### Sociodemographic variables

Sociodemographic variables will include age (in years), gender (male; female; diverse), highest level of education (no professional qualification; recognized professional training; bachelor’s degree or equivalent; master’s degree or equivalent; doctorate), employment status (student; employed full-time; employed part-time; employed on a marginal basis (no more than 520€/month); unemployed; retirement pension; disability pension; homemaker; educational retraining; reintegration program; other), household income (in EUR), marital/relationship status (in a partnership living together; in a partnership not living together; single, divorced or separated; widowed), number of children. In addition, participant characteristics will include height (in cm), weight (in kg), pregnancy, previous meditation experience (yes or no), and openness to meditation (yes or no).

#### Primary and secondary outcomes

##### Primary outcome

The primary outcome will be self-reported 7-day point prevalence abstinence from smoking (PPA) at t_3_ and t_4_, operationalized as not having smoked any cigarettes, not even a single puff, in the last 7 days [62].

##### Secondary outcomes

Smoking cessation related secondary outcomes at t_3_ and t_4_ will include percentage reduction in CPD, 30-days PPA, repeated PPA, continuous PPA (< 5 cigarettes since target quit date), and urges to smoke (VRS v4-1) [63]. Other secondary outcomes will include perceived stress (Perceived Stress Scale; PSS-10) [64], mindfulness (Freiburg Mindfulness Inventory short version; FFA) [65], smoking self-efficacy (“How confident are you that you will be completely abstinent from smoking cigarettes in one year from now? (1 “not confident at all” -10 “very confident”), and Smoking Self-Efficacy Questionnaire; SEQ-12) [66], subjective health (Short Form Health Survey; SF-12) [67], and life satisfaction (L-1) [68].

The acceptability of the nuumi program will be operationalized as (1) usefulness in quitting smoking ("How helpful do you find the program in not smoking cigarettes?", "To what extent does the program increase your confidence to quit smoking?”), (2) satisfaction with the program ("How would you rate your overall satisfaction with the smoking cessation program?"/"How likely are you to recommend the program to friends or colleagues who want to quit smoking?"), (3) comprehensibility of the content ("How informative did you find the content of the behavioral training?” / "How understandable did you find the content of the behavioral training?”). Usability of the app is also assessed using the System Usability Scale (SUS) [69].

For each questionnaire, the validated German version will be used. If no German version is available, translations will be made by the study staff.

#### Other variables

##### Smoking behavior-related variables

Smoking behavior-related variables include years of smoking, motivation to quit (Motivation To Stop Scale) [70], number and method(s) of previous quit attempts, use of alternative tobacco products and/or ECs, current participation in other smoking cessation program, current use of NRT, and withdrawal symptoms (Wisconsin Smoking Withdrawal Scale; WSWS2-B) [71]. Additionally, EC dependence is queried (Penn State Electronic Cigarette Dependence Index; PSECDI) [72].

##### Adherence and engagement

Adherence is operationalized as (1) self-reported use of the EC (current use and number of days of use), and (2) self-reported engagement with the app (number of daily/weekly uses, number of modules completed, minutes meditated). Participants will be asked whether they participate in any other smoking cessation program in each survey from t_0_ onwards.

##### Subjective evaluation and side effects

Subjective evaluation of the EC and side effects will be measured using items from a variety of scales assessing subjective effects of ECs [12]. Participants will be asked about adverse events in each survey from t_1_ onwards and requested to report all adverse events occurring between the survey time points to the study staff.

##### Nuumi program feature utilization

In addition to the self-reported EC and app utilization data, app feature utilization data of the nuumi program will be collected by the manufacturer of nuumi, Sanos Group GmbH, for the first 8 weeks after program initiation. Using this data, separate secondary analyses will be conducted to investigate the degree to which users engaged with the individual program features, and the relationships between utilization of individual features and smoking abstinence at 4 weeks and 8 weeks post-baseline. Utilization metrics collected by Sanos Group GmbH will include (1) the number of behavioral therapy lessons completed, (2) the number of meditations completed and/or repeated, (3) the number of toolbox exercises completed, (4) the number of times the progress tracking feature was accessed, and (5) the number of days the EC was used.

##### Sample size

Formal sample size calculations are not required for single-arm pilot studies [73]. Target sample size estimation is based on single-arm pilot studies evaluating interventions for smoking cessation [74–76]. In order to address the primary and secondary aims of this trial, and to account for attrition, the target sample size is 70 participants.

### Statistical methods

#### Quantitative data analyses

Analyses will be conducted using the statistical software R, version 4.4.0 (R Core Team, 2024). Given the exploratory nature of this study, data analyses will primarily be descriptive. For categorical variables, frequencies and percentages will be reported. For continuous variables, mean and standard deviation (SD) will be reported. If applicable, statistical comparisons for paired data will be applied to compare scores observed before and after program participation. Statistical significance will be evaluated using a repeated measures two-tailed t-test; the alpha level will be set at 0.05 and a 95% confidence interval will be used. Intention-to-treat (ITT) analyses will be conducted, assuming all individuals not completing a follow-up survey have resumed smoking. In addition, complete cases will be analyzed, i.e. only participants completing the t_3_ and t_4_ survey, respectively, will be included in these analyses. Percentages and patterns of missing data will be assessed using Little’s Missing Completely At Random test and adequate data imputation techniques will be used, if applicable.

##### Primary and secondary outcomes

For the primary outcome, 7-day PPA at t_3_ and t_4_, number and percentage of participants self-reporting abstinence will be reported. For the secondary outcomes, measures of frequency and percentages will be reported for 30-days PPA, repeated PPA and continuous PPA, and measures of central tendency will be reported for CPD and urges to smoke. Paired t-tests will be conducted to assess changes in CPD and urges to smoke from t_0_ to t_3_ and from t_0_ to t_4_. Mean and SD will be reported for perceived stress, mindfulness, smoking self-efficacy, subjective health and life satisfaction, and paired t-tests will be conducted to assess within-subject changes in these variables. Acceptability outcomes will be reported descriptively.

##### Participant characteristics

Frequency measures and percentages will be reported for categorical variables (gender, education, occupational status, household income, relationship status, previous meditation experience, openness to meditation). For continuous variables (age, number of children, height, weight), mean and SD will be reported. Changes in weight will be calculated using paired t-tests. In addition, it will be investigated how demographic characteristics of the participants are associated with the collected outcomes using bivariate tests, including Pearson’s r correlations.

##### Other variables

Frequency measures and percentages will be reported for method(s) of previous quit attempts, use of alternative tobacco products and ECs, participation in other smoking cessation programs, NRT use, and EC dependence. Mean and SD will be calculated for years of smoking, motivation to quit, number of previous quit attempts, and withdrawal symptoms. To assess adherence and engagement, frequencies and percentages of current EC use, and number of days of use will be reported. Mean and SD for engagement with the app will be calculated. Further, frequencies and percentages will be reported for the subjective evaluation of the EC, side effects, and adverse events.

##### Nuumi program feature utilization

As indicators for feature utilization, we will analyze a set of variables including (1) the number of completed behavioral therapy lessons, (2) the number of meditation audios completed and/or repeated, (3) the number of toolbox exercises completed and/or repeated, (4) the number of times the progress tracking feature was accessed, and (5) the number of days the nuumi EC was used. A total engagement score will be calculated across all intervention components. For each of the five utilization variables, a median split will be conducted, each resulting in two categories of 0 (“below median utilization”) and 1 (“above median utilization”). The five scores will be added, resulting in a single score (ranging from 0 to 5). Two logistic regressions will be performed with total program engagement as the predictor variables and 7-day PPA from smoking at 4-weeks and 8-weeks post-baseline as the dependent variables (with 0 indicating “non-abstinent” and 1 indicating “abstinent”). To examine the relationship between feature utilization and smoking cessation, a total of 10 univariate logistic regressions for each of the five features will be performed to predict 7-day PPA from smoking at 4-weeks and 8-weeks post-baseline. The predictor variable will be the respective app feature utilization, and 7-day PPA will be the dependent variable.

To control for confounding effects on the relationship between feature utilization and smoking cessation outcomes, we will control for baseline characteristics that significantly differed by smoking status at t_1_, or t_2_, or both. To identify these variables, independent samples t-tests will be conducted for continuous variables, and chi-square tests for categorical variables.

#### Qualitative data analyses

The 1:1 semi-structured interviews will be conducted by research staff, recorded via Zoom and transcribed verbatim. Interview guidelines were developed to ensure all relevant information to answer the research questions is collected and comparability between interviews is guaranteed. The recordings will be analyzed using qualitative content analysis [77]. The primary analysis will be carried out by HS and CH using MAXQDA (Verbi GmbH, Berlin, Germany). After reading and re-reading the transcripts, the content will be split into meaningful categories. An initial coding scheme will be developed individually by HS and CH by assigning codes to the categories based on similarities. Codes will be compared, and discrepancies will be discussed between HS and CH until consensus is achieved. While applying the agreed coding scheme, HS and CH will repeatedly check for consistency by independently coding some same segments of text and then comparing results. Overarching themes that emerge from the identified categories will be identified and quotes from the interviews illustrating each theme will be selected.

Anonymized transcripts of the audio recordings will be stored in a secure university network location accessible only by authorized members of the research team. Once the audio recordings have been transcribed, the audio recordings will be deleted.

### Ethical considerations

#### Research ethics approval

Ethics approval was obtained from the Institutional Review Board (IRB) in September 2023 (123/2023). Any amendments will be submitted to the IRB, to the study funder, to the German Clinical Trial Registry, and to the journal where this manuscript was submitted.

#### Confidentiality and access to data

Personal information about potential and enrolled participants will be collected only by members of the research team and cannot be accessed by other individuals. Personal information and survey data will be pseudonymized using an identification number. Only authorized study staff will have access to any of the study data. Participant files will be stored for a period of 10 years after completion of the study in anonymized format.

## Discussion

### Expected results

This study will evaluate the initial efficacy, acceptability, and psychological outcomes of a digital EC-supported smoking cessation intervention in a real-world setting. Anticipated date of study completion is June 2024; we expect for results to be available in August 2024. The findings of the study will provide valuable insights into the design and implementation of future digital smoking cessation interventions for adults who smoke and are motivated to quit smoking. The study will further provide findings informing the design and implementation of a future RCT evaluating the effectiveness and efficacy of the intervention.

### Strengths and limitations

Several strengths are associated with the present study. To the best of our knowledge, the present study is the first trial to evaluate a smoking cessation program featuring an integrated EC- and app-based behavioral therapy developed based on comprehensive scientific evidence in a real-world setting. Providing data on feasibility, acceptability and initial efficacy may inform further development of similar interventions. The study results will be documented in accordance with international documentation guidelines, including an adapted CONSORT-EHEALTH diagram for pilot and feasibility trials [52, 53].

The research questions of this trial will be assessed using data collected at 12 weeks and 24 weeks post-baseline. This follow-up period is sufficient to detect relapse, as relapse occurs most often during the first few weeks after a quit attempt [78].

Another strength of this study is the mixed-methods approach which allows for a comprehensive understanding of the feasibility and acceptability of nuumi. These insights will contribute to providing a more complete picture of the utility of EC-supported, app-based smoking cessation, which in turn may inform future developments of mHealth-supported EC-based smoking cessation interventions.

We acknowledge that this study design has several limitations. First, as it is designed as a single-arm trial without a control group, the external and internal validity of the results are limited. However, single-arm studies are a common design used to test the feasibility, acceptability, and initial efficacy of new interventions and to pilot study procedures for future RCTs [79].

Although we will not directly compare nuumi with a similar intervention or TAU, descriptive results of initial efficacy, acceptability and user engagement can serve as preliminary indicators of comparability with other smoking cessation interventions. While a sample size of 70 participants does not allow for generalization of findings to a broader population, our sample is larger than those usually included in pilot and feasibility trials [73].

Second, participants in this study will be recruited by online advertisements run by the manufacturer of nuumi, Sanos Group GmbH, which presents a risk of selection bias, limiting the generalizability of the results. We have taken steps to reduce the risk of bias by ensuring that recruitment website content will be written, and eligibility screening will be conducted by research staff, limiting the role of the manufacturing company as much as possible.

A third limitation comes with the inclusion criterion of only including individuals motivated to quit smoking, leading to a group of adults who smoke who differ from the general population of smokers. Moreover, individuals with severe psychiatric or physical illness are excluded from the study. Considering the high prevalence of smoking in populations with severe health conditions, these exclusion criteria limit the generalizability of the results to this population and the opportunity is missed to assess the intervention in this vulnerable group.

Fourth, given the novelty of the intervention and the specific, integrated features of nuumi, the external validity of the results of this trial to the diverse group of commercially available ECs will be limited. Additionally, the nicotine delivery of the EC used in the program has not been tested (e.g. through in-lab studies) and it is unclear if users can derive sufficient nicotine from the EC to suppress their cravings. If an EC is not able to suppress cravings effectively, cigarette smoking may continue [80, 81].

Fifth, in this pilot study, we will rely only on self-reported abstinence rates in assessing smoking abstinence rates. This practice goes in line with expert consensus suggesting that biochemical validation of self-reported abstinence is not necessary in studies where data is collected over the web, and that the levels of misrepresentation are generally low [82]. However, CO and cotinine verification of abstinence rates will be conducted in an RCT to be conducted after completion of the pilot study.

Finally, this pilot trial aims to investigate nuumi in its entirety, and no additional testing will be conducted to investigate feasibility and/or effectiveness of isolated program features (i.e. EC and app features). Further research will be needed to investigate the singular impact of the program components. In the framework of the RCT set to be conducted after the pilot study, extensive app data will be collected on single program feature usage. Using this data, it will be determined whether and how single features of the intervention are associated with successful smoking cessation.

Leveraging the opportunity to collect extensive app behavior data from trial participants and app users, the authors of this paper will collaborate with the manufacturing company of nuumi to refine the intervention further. Specifically, it is planned to develop an overarching behavior change feature in the form of a comprehensive *nuumi score* (NUS) into the app that integrates data on users’ progress across all behavior tracked in the app, i.e. EC puffing behavior, nicotine reduction, completion of behavioral therapy modules, meditation library usage, and self-efficacy ratings. The NUS aims to show individuals their progress on their behavior change path in all parts of the nuumi intervention in the form of a comprehensive, single score. By integrating progress tracking of all strains of therapy where progress can be achieved, the NUS allows individuals to be rewarded for overall progress even when only little or no progress is made in some areas. In this way, the NUS is hypothesized to serve as positive reinforcement, activating positive affect as well as enhancing reward expectation and motivation. A paper on the development and implementation of the NUS is currently being prepared for publication by the authors.

## Conclusions

Smoking remains a leading cause of preventable diseases, including cancer, heart disease, and respiratory conditions, placing a heavy burden on healthcare systems and society. Reducing smoking rates is of paramount importance for both individual and public health. Implementing effective novel smoking cessation interventions, as explored in this research, is of imperative importance to create a healthier and more prosperous future for generations to come. Within a theoretical framework drawing from the BCRM, nuumi presents a novel, integrated smoking cessation intervention enabling adults who smoke who do not want to use methods recommended by medical guidelines or who could not achieve success with these methods. The intervention addresses both the physical and psychological aspects of tobacco addiction. The physical aspects are addressed by an EC, which is initially used for smoking cessation and, after successful smoking cessation, is to be discontinued completely by steadily reducing the nicotine concentration in the liquid, so that the person uses neither cigarettes nor ECs at the end of the program. The app-based behavioral therapy supports individuals in this process by modifying thoughts, beliefs, and behaviors associated with smoking. Thorough evaluation of acceptability and feasibility of the intervention in the respective target population is imperative. The assessment holds significant value as it informs development and implementation of similar mHealth interventions and guides future research. Utilizing a mixed methods design, the present study seeks to produce findings including smoking cessation, acceptability, and feasibility of a digital EC-supported smoking cessation intervention.

## Data Availability

Not applicable.

## Appendix A

See Tables 1 and 2

**Table 1:** Overview of the content of the 11 modules included in the nuumi behavioral therapy

Module	Educational content	Exercises
Fundamentals	Psychoeducational content on the mechanisms of cigarette dependence is provided. Nuumi use guidelines as well as technological and safety aspects of EC use are discussed. Content supporting the transition from cigarettes to the EC is featured e.g. (implementation intentions [83], social support)	- Formulation of if–then plans [83] aiming at using the EC instead of cigarettes - Preparation of physical and social environment for quitting cigarette use (removing cigarettes and physical triggers; informing social environment and asking for support)
1. Habits and addiction	In-depth information is provided on formation and maintenance of habits and addiction, explaining the role of nicotine and the dopaminergic reward system, supporting individuals in gaining a deeper understanding of their addiction as a basis for learning effective strategies to overcome it	- Reflection of daily habits, including rating them (beneficial, non-beneficial, neutral) - Reflection of personal smoking history - Motivational interview: assessment of participants’ preparedness for quitting, individual motivations for quitting smoking, and the advantages and disadvantages of continuing to smoke versus quitting
2. The way out of addiction	Participants learn about effective, positive goal setting in health behavior change [60] and are prompted to formulate their individual goal for the nuumi program. Participants learn about the different stages and principles of behavior change and the life-long learning capacity of the human brain (i.e. neuroplasticity)	- Formulation of a positively framed individual goal for the program (e.g. “I want to be a non-smoker.”, “I want to live a dependence-free live”) - Quiz - Reflection of novel and positive experiences in the past days
3. Stress	Psychoeducation on stress and its role in smoking cessation and relapse is provided. Participants learn what the short-term and long-term consequences of stress can be and how they can recognize stress in themselves	- Assessment of current stress level - Exploration of individual stress warning signals - Guided body scan meditation - Reflection of novel and positive experiences in the past days
4. Relaxation	Content on the role of relaxation as an antagonist of stress is provided. Participants are taught to differentiate between active and passive methods of relaxation. Information is provided regarding the positive effects of regular relaxation practice on focus, emotion regulation, body awareness, sleep, and self-regulation	- Diaphragmic breathing - Breathing meditation - *SBRC technique* (stop—breathe—reflect—choose) - Reflection of novel and positive experiences in the past days
5. Mindfulness	Psychoeducation on mindfulness (formal versus informal) and an introduction to meditation is provided. Participants are prompted to practice guided mindful walking and learn about the benefits of mindfulness for smoking cessation and relapse prevention. Individuals are asked to identify their triggers for smoking/use of EC and learn to practice awareness when encountering relapse triggers in their daily life	- *Five senses* exercise as a coping strategy to stop rumination in demanding situations during withdrawal or stressful situations - Mindful walking meditation - Quiz - Identification of smoking/EC use triggers - Reflecting on novel and positive experiences in the past days
6. Thoughts	Information on thoughts, mental distortions and maladaptive coping mechanisms (e.g. smoking) is provided. Exercises target identification of irrational and maladaptive thought patterns about, among other topics, smoking (e.g. “I will never be able to enjoy life anymore without smoking.”) and teach coping strategies like cognitive restructuring, and thought labelling	- Thought labelling - Practice of questioning thoughts critically - Reflection of novel and positive experiences in the past days
7. Emotions	Psychoeducational content features information on adaptive and maladaptive emotion regulation strategies. Participants develop an understanding about how emotions affect smoking cravings and behavior and learn to employ cognitive restructuring and labelling techniques to cope with negative affect. Participants are educated about self-compassion, are invited to reflect about their own degree of self-compassion, and learn strategies to increase this skill	- Emotion labelling - *Urge surfing* [57] to cope with negative mood and smoking cravings - *Pause button*; an exercise to learn taking breaks to mindfully recognize emotions - Reflection of novel and positive experiences in the past days
8. Nutrition and exercise	Participants are introduced to the concepts of mindful and healthy eating and learn how to manage food cravings. Physical exercise is emphasized as an effective strategy for overcoming cravings and for weight control after quitting smoking. Participants are further educated about the pitfalls of alcohol in smoking cessation and are introduced to coping strategies	- SBRC technique is repeated - Visualization of obstacles for eating healthy and exercising regularly and formulating if–then plans for how to overcome these obstacles - Reflection of novel and positive experiences in the past days
9. Handling relapse	Relapse prevention content consists of educating participants about the role and effects of negative thought spirals after relapse. Participants are advised to see relapses as temporary setbacks, and are encouraged to avoid blaming themselves. Coping strategies like urge surfing and SBRC technique are repeated. Also, the role of social support in successful relapse prevention is highlighted	- Assessment of participants’ preparedness for quitting using also the EC, and re-assessment of individual motivations for quitting smoking/EC use - *Safety net*: Participants are asked to identify people in their environment to support them on their relapse prevention journey - Mountain meditation as a tool for remaining calm during stressful situations, e.g. when cravings occur - Reflection of novel and positive experiences in the past days
10. Your New Me	Participants’ stress levels are re-evaluated, and they are prompted to reflect on their stress warning signals and coping skills, as well as to reflect on potential changes that occurred in the span of the course. Coping strategies such as practicing mindfulness when encountering triggers, urge surfing, SBRC technique, Five Senses, and breathing meditation are repeated, and implementation intentions [83] are recommended to be used to implement the adoption of these coping strategies into daily life	- Visualization exercise to identify future situations which could potentially lead to relapse and learn to prepare for such situations - Reflection of the nuumi program

**Table 2:** Overview of schedule of enrollment, intervention and assessments

	Study period					
Timepoint	Enroll-ment	Baseline	4 weeks post-baseline	8 weeks post-baseline	12 weeks post-baseline	24 weeks post-baseline
	− t_1_	t_0_	t_1_	t_2_	t_3_	t_4_
Enrollment:						
Eligibility screen	✓					
Informed consent	✓					
Intervention:		✓	✓	✓	✓^a^	
Assessments:						
Participants ‘ characteristics						
Age	✓	✓				
Gender	✓	✓				
Height		✓				
Weight		✓	✓	✓	✓	✓
Pregnancy	✓	✓	✓	✓	✓	✓
Primary outcome						
7-day PPA			✓	✓	✓	✓
Secondary outcomes						
CPD	✓	✓	✓	✓	✓	✓
30-days PPA						
Repeated PPA			✓	✓	✓	✓
Continuous PPA			✓	✓	✓	✓
Urges to smoke		✓	✓	✓	✓	✓
Perceived stress		✓	✓	✓	✓	✓
Mindfulness		✓	✓	✓	✓	✓
Smoking self-efficacy		✓	✓	✓	✓	✓
Subjective health		✓	✓	✓	✓	✓
Life satisfaction		✓	✓	✓	✓	✓
Acceptability		✓	✓	✓	✓	✓
Usability		✓	✓	✓	✓	✓
Adherence			✓	✓	✓	✓
Other variables						
Nicotine dependence		✓	✓	✓	✓	✓
Use of alternative tobacco products	✓	✓	✓	✓	✓	✓
Years of smoking	✓	✓				
Motivation to stop smoking	✓					
Quit attempts and method(s)		✓				
Quit date			✓			
Withdrawal symptoms		✓	✓	✓	✓	✓
EC use	✓	✓	✓	✓	✓	✓
NRT use	✓	✓	✓	✓	✓	✓
Participation in another smoking cessation program	✓	✓	✓	✓	✓	✓
Adverse events			✓	✓	✓	✓
EC dependence			✓	✓	✓	✓
Subjective evaluation and side effects of EC			✓	✓	✓	✓
Dropout			✓	✓	✓	✓
Meditation experience & openness towards meditation		✓				

^a^Participants receive unlimited access to the nuumi app; they can use the nuumi EC as long as they have pods (minimum 12 weeks)

## Appendix B

### Informed consent forms

#### Informed consent form study participation

Dear interested study participant,

You have received an information letter about the nuumi feasibility study by email. In this document you have received important information about this project.

If you have read the document carefully and completely, you can now consent to study participation in this survey.

If you have not yet read the document, please do so now. Enrolment in the study can only begin if you consent to participate.

If you have any questions, please email nuumi@uni-wh.de.

Yours sincerely.

Prof. Dr. med. Tobias Esch and the project team.

#### Personal details

Please fill in your personal details here. You can only register for the study if you provide complete information here. Please note that non-truthful information may lead to subsequent exclusion from the study.

First name: _______.

Surname: _______.

Date of birth: _______.

Email address: _______.

Place of residence: _______.

#### Declaration of consent to participate in the “Nuumi Feasibility Study” project

This part refers to the information letter you received by email.

- I have received, read and understood the information letter for participants with information on data protection.
- I have been informed about the content and aim of the evaluation of the project "Nuumi Feasibility Study". The evaluation is being conducted under the direction of Prof. Dr. Tobias Esch at the University of Witten/Herdecke. By agreeing to participate in the evaluation, I would like to support the scientific project.
- I agree that I will be invited to participate in an online survey by email at five points in time.
- I agree that I may be invited to participate in a telephone or video interview at two points in time.
- I am aware that all research data will be analyzed anonymously. Publication will take place in anonymized form so that it is no longer possible to draw conclusions about my person.
- I am aware of my rights with regard to the storage of my personal data in accordance with the General Data Protection Regulation (DSGVO).
- My participation in the evaluation is voluntary. I know that I can withdraw my consent to participate at any time, verbally or in writing and without giving reasons, without any disadvantage to me.
- I was comprehensively informed about my rights and about the time required for me as a participant. All my questions were answered comprehensibly and in full.
- I agree with all the above statements and hereby consent to participate in the study.

___ Yes___ No

### Informed consent form qualitative interview participation

Dear study participant,

You have received an information letter by email regarding the individual interview as part of the Nuumi feasibility study.

If you have read the document carefully and completely, you can now consent to participate in the interview as part of this survey.

If you have any questions, please email nuumi@uni-wh.de.

Yours sincerely.

Prof. Dr. med. Tobias Esch and the project team.

#### Personal details

Please fill in your personal details here. You can only register for the study if you provide complete information here. Please note that non-truthful information may lead to subsequent exclusion from the study.

First name: _______.

Surname: _______.

Date of birth: _______.

Email address: _______.

Place of residence: _______.

#### Declaration of consent to participate in the qualitative interview within the "Nuumi Feasibility Study" project

- I was informed about the content and aim of the evaluation of the individual interview. The evaluation is being conducted under the direction of Prof. Dr. Tobias Esch at the University of Witten/Herdecke. I have received, read and understood a letter of information for participants with information on data protection.
- I agree to be invited to an individual interview via video phone call by email.
- I am aware that all research data will be analysed anonymously. Publication will take place in anonymised form so that it is no longer possible to draw conclusions about my person. I am aware of my rights with regard to the storage of my personal data in accordance with the General Data Protection Regulation (DSGVO). My participation in the evaluation is voluntary. I know that I can withdraw my consent to participate at any time, verbally or in writing and without giving reasons, without any disadvantage to me.
- I was comprehensively informed about my rights and also about the time required for me as a participant. All my questions were answered comprehensibly and in full. Under these conditions, I agree to participate in the individual interview.

___ Yes___ No.

## Abbreviations

BCRM: Behavior Change Resource Model
BCT: Behavior change technique
CBT: Cognitive-behavioral therapy
CPD: Cigarettes per day
EC: Electronic cigarette
ITT: Intention-to-treat
mHealth: Mobile health
MII: Mindfulness-informed intervention
NUS: Nuumi score
RCT: Randomized controlled trial
SD: Standard deviation
TAU: Treatment as usual
